# Supplementary material for: Investigating Gender-based violence against internally displaced women in Debre Berhan, Central Ethiopia: A mixed-methods study using the socio-ecological framework
Source: PLoS One. 2025 Aug 13;20(8):e0329840. doi: 10.1371/journal.pone.0329840 (PMC12349714; doi:10.1371/journal.pone.0329840)
Supplement: S1 File — (DOCX) [file pone.0329840.s001.docx]

**Supplementary** file 1: English Version Questionnaire

Part I: Socio-demographic and reproductive characteristics of respondents

| No | Question | Answer | Skip |
| --- | --- | --- | --- |
| 1 | How old are you (age in completed years) | ------- completed years |  |
| 2 | What is your current marital status? | 1. Married 2. Single 3. Separated 4. Divorced 5. Sexual union |  |
| 3 | How many children born alive do you have? | --------no of children |  |
| 4 | What is the highest level of education you attained? | 1. Unable to read and write 2. Able to read and write (but not attended formal education) 3. Primary (1-8) 4. Secondary (9-12) 5. Post-secondary (above 12) |  |
| 5 | Have you ever used family planning? | 1. Yes 2. No |  |
| 6 | Are you currently using any of the family planning methods? | 1. Yes 2. No | If the answer is option 2(no), skip to 8 |
| 7 | If yes to question 6, what type of family planning method? | 1. Implanon 2. IUD 3. Pills 4. Injectables 5. Condoms 6. Others (specify it) …………… |  |

Part 2: GBV types, perpetrators and time when it occurred

| No | Question | Answer | Skip |
| --- | --- | --- | --- |
| 8 | Have you ever been threatened with physical or sexual violence by someone in your home or outside of your home? | 1. Yes 2. No | If the answer is option 2 (no), skip to question 12 |
| 9 | When did the threatened physical or sexual violence occur? More than one answer is possible | 1. In my home (before migration) 2. During migration/transit 3. At the camp |  |
| 10 | Did the threatened physical or sexual violence occur in the past 12 months? | 1. Yes 2. No | If the answer is option 2 (no), skip to question 12 |
| 11 | Who was the perpetrator of the threatened physical or sexual violence that occurred in the past 12 months? More than one answer is possible | 1. Intimate Partner/ husband 2. Family 3. Police 4. Soldier 5. Neighbour 6. Camp Guard 7. Others*(please specify) |  |
| 12 | Have you ever been hit, punched, kicked, slapped, choked, hurt with a weapon, or otherwise physically hurt by someone in your home or outside of your house? | 1. Yes 2. No | If the answer is option 2 (no), skip to question 16 |
| 13 | When did this physical violence occur? More than one answer is possible | 1. In my home (before migration) 2. During migration/transit 3. At the camp |  |
| 14 | Did this physical violence occur in the past 12 months? | 1. Yes 2. No | If the answer is option 2 (no), skip to question 16 |
| 15 | Who was the perpetrator of this physical violence that occurred in the past 12 months? More than one answer is possible | 1. Intimate Partner/ husband 2. Family 3. Police 4. Soldier 5. Neighbour 6. Camp Guard 7. Others*(please specify) |  |
| 16 | Have you ever been forced to have sex against your will? | 1. Yes 2. No | If the answer is option 2 (no), skip to question 20 |
| 17 | When did this sexual violence occur? More than one answer is possible | 1. In my home (before migration) 2. During migration/transit 3. At the camp |  |
| 18 | Did this sexual violence occur in the past 12 months? | 1. Yes 2. No | If the answer is option 2 (no), skip to question 20 |
| 19 | Who was the perpetrator of this sexual violence that occurred in the past 12 months? More than one answer is possible | 1. Intimate Partner/ husband 2. Family 3. Police 4. Soldier 5. Neighbour 6. Camp Guard 7. Others*(please specify) |  |
| 20 | Have you ever been forced to have sex to be able to eat, have shelter, or have sex for essential services (such as protection or school) because you or someone in your family would be in physical danger if you refused? | 1. Yes 2. No | If the answer is option 2 (no), skip to question 24 |
| 21 | When did this forced sex occur? More than one answer is possible | 1. In my home (before migration) 2. During migration/transit 3. At the camp |  |
| 22 | Did this forced sex occur in the past 12 months? | 1. Yes 2. No | If the answer is option 2 (no), skip to question 24 |
| 23 | Who was the perpetrator of this forced sex that occurred in the past 12 months? More than one answer is possible | 1. Intimate Partner/ husband 2. Family 3. Police 4. Soldier 5. Neighbour 6. Camp Guard 7. Others*(please specify) |  |
| 24 | Have you ever been physically forced or made to feel that you had to become pregnant against your will? | 1. Yes 2. No | If the answer is option 2 (no), skip to question 28 |
| 25 | When did this forced pregnancy occur? More than one answer is possible | 1. In my home (before migration) 2. During migration/transit 3. At the camp |  |
| 26 | Did this forced pregnancy occur in the past 12 months? | 1. Yes 2. No | If the answer is option 2 (no), skip to question 28 |
| 27 | Who was the perpetrator of this forced pregnancy that occurred in the past 12 months? More than one answer is possible | 1. Intimate Partner/ husband 2. Family 3. Police 4. Soldier 5. Neighbour 6. Camp Guard 7. Others*(please specify) |  |
| 28 | Have you ever been coerced or forced into marriage? | 1. Yes 2. No | If the answer is option 2, it is the end, start the second participant interview |
| 29 | When did this forced marriage occur? More than one answer is possible | 1. In my home (before migration) 2. During migration/transit 3. At the camp |  |
| 30 | Did this forced marriage occur in the past 12 months? | 1. Yes 2. No |  |
| 31 | Who was the perpetrator of this forced marriage that occurred in the past 12 months? More than one answer is possible | 1. Intimate Partner/ husband 2. Family 3. Police 4. Soldier 5. Neighbour 6. Camp Guard 7. Others*(please specify) |  |
