# Supplementary material for: Investigating Gender-based violence against internally displaced women in Debre Berhan, Central Ethiopia: A mixed-methods study using the socio-ecological framework
Source: PLoS One. 2025 Aug 13;20(8):e0329840. doi: 10.1371/journal.pone.0329840 (PMC12349714; doi:10.1371/journal.pone.0329840)
Supplement: S2 File — (DOCX) [file pone.0329840.s002.docx]

**Supplementary file 2: In-depth interview guideline for GBV survivors**

**Background characteristics**

Age_________

Sex_________

Education status _________

Marital status_________

Have children_________

No children _________

1. How do internally displaced women who survived gender-based violence access services?

**Probing points:**

- How do they access medical health services?
- How do they access legal services?
- How do they access psychosocial support services?

2. Why do internally displaced women who survived gender-based violence not access/utilise GBV services?

**Probing points:**

• How do you describe the barriers to accessing GBV services?

• How do you see barriers from the following sides

o Individual levels (fears, perceptions, attitudes, knowledge on GBV)

o Community perspectives (support, attitude, knowledge and norms)

o Institutional /health facility perspectives (accessibility, quality, affordability)

o Structural issues (camp context, security, legal issues, policy issues)

3. How do you describe the facilitators for GBV survivors to access care and support?

**Probing points:**

• How do you see barriers from the following sides

o Individual levels (attitude, knowledge, self-confidence)

o Community perspectives (support, attitude, knowledge and norms)

o Institutional /health facility perspectives (accessibility, quality, affordability)

o Structural issues (camp context, security, legal issues, policy issues)
