# Supplementary material for: Investigating Gender-based violence against internally displaced women in Debre Berhan, Central Ethiopia: A mixed-methods study using the socio-ecological framework
Source: PLoS One. 2025 Aug 13;20(8):e0329840. doi: 10.1371/journal.pone.0329840 (PMC12349714; doi:10.1371/journal.pone.0329840)
Supplement: S3 File — (DOCX) [file pone.0329840.s003.docx]

**Supplementary 3: In-depth interview guideline for Key informants**

**Background characteristics**

Age_________

Sex_________

Education status _________

Marital status_________

Years of experience _________

Organization currently working for_________

1. How do internally displaced women who have experienced gender-based violence (GBV) access services in Deberbrhan

Probing Points

• Which services are available?

• How are survivors referred or linked to available services?

• What role does your institution play in the referral or care process?

• How timely and suitable is the response for survivors?

2. What are the primary impediments to survivors getting GBV services, according to your experience?

Probing points:

• Individual-level issues include fear, humiliation, and lack of awareness.

• Barriers at the community level include societal norms, stigma, and a lack of support.

• Institutional obstacles, including personnel capacity, resources, and infrastructure.

• Structural constraints include security concerns, camp conditions, and legal/policy deficiencies.

3. What variables promote access to GBV services for internally displaced women?

Probing points:

- Individual-level: Survivor's knowledge, positive attitude, and self-confidence.
- Community-level: supportive norms, community awareness, and peer support.
- Institutional level: skilled people, accessible services, and effective referral
- Structural level: Supportive policies, camp security, and actor coordination
