# Supplementary material for: Investigating Gender-based violence against internally displaced women in Debre Berhan, Central Ethiopia: A mixed-methods study using the socio-ecological framework
Source: PLoS One. 2025 Aug 13;20(8):e0329840. doi: 10.1371/journal.pone.0329840 (PMC12349714; doi:10.1371/journal.pone.0329840)
Supplement: S4 File — (DOCX) [file pone.0329840.s004.docx]

**Supplementary 4 The final modified code book**

| **Code name** | **Code definition** | **When to use** | **When not to use** | **Example** |
| --- | --- | --- | --- | --- |
| Reluctance to disclose | Texts highlighting that GBV survivors are reluctant to disclose their experiences to anyone. | Texts highlighting that GBV survivors are reluctant to disclose their experiences to anyone, including family members, health workers, or legal authorities. |  | *"Most of the time, we, as survivors, don’t clearly explain our situation to the relevant authorities."* |
| Self-isolation | Texts indicate that GBV survivors tend to isolate themselves as a result of the violence | Apply this code to texts mentioning that GBV survivors tend to isolate themselves due to the violence. | Do not apply this code to texts that mention the community isolating survivors, as there is a separate code for that. | *"Because I didn’t want to integrate with others, I chose not to share my condition with them."* |
| Lack of awareness | Texts mentioning that GBV survivors and/or the general camp community lack awareness about the available services for survivors. | Apply this code to texts mentioning that GBV survivors and/or the general camp community lack awareness about available services, such as healthcare, legal, and psychosocial support for survivors |  | *"I haven't seen or heard that healthcare is provided to GBV survivors."* |
| Normalisation of violence | Texts describing that GBV survivors or the general camp community perceive any form of GBV as normal | Apply this code to texts describing that GBV survivors or the general camp community perceive any form of GBV as normal |  | *"Women themselves ask, 'Why did they [police/prosecutors] question him [the perpetrator] when we love him?'* |
| Self-efficacy | Texts describe that GBV survivors have the confidence to report the abuse and seek care and support on their own | Apply this code to texts mentioning that GBV survivors have the confidence to report the abuse and seek care and support on their own |  | *"With confidence in myself, I decided to go to the health facility"* |
| Self-blame | Texts describe that survivors often feel self-blame, believing they are responsible for the violence. | Apply this code to texts mentioning that GBV survivors believe they are responsible for the violence. | "Don’t apply this code to texts describing that the blame is not self-blame, but rather from the community." | "*She may not go to the court if it is her fault"* |
| Fear of gossip and stigma | Texts mention that GBV survivors fear gossip and stigma, and as a result, don’t seek care. | Apply this code to texts mentioning that GBV survivors fear gossip and stigma, and as a result, don’t seek care. |  | *"Even if we refer her to the hospital or legal services, she might refuse because she believes others are pointing fingers at her."* |
| Fear of retaliation | Texts describing that survivors fear reprisals or attacks by the perpetrator. | Apply this code to texts describing survivors who fear reprisals or attacks by the perpetrator. |  | *"She fears the perpetrator may cause her further serious injury if she goes to court."* |
| Fear of family disintegration | Texts mention that GBV survivors fear family disintegration as a result of exposing their status. | Apply this code to texts mentioning that GBV survivors fear family disintegration as a result of exposing their status, such as fearing marriage dissolution, family separation, etc. |  | *"The first concern is that they fear their husband might not believe them and may divorce them. They worry this could lead to disengagement from the marriage.* |
| Support from NGOs and volunteers | Texts describe that NGOs and volunteers in camps play a key role in addressing GBV and promoting access to care. | Apply this code to texts describing that NGOs and camp volunteers play a key role in addressing GBV and promoting access to care. |  | *"Some organizations are advocating for women’s rights. Currently, some are working on the prevention of GBV."* |
| Availability of awareness creation activities | Texts describing that awareness-creation activities on GBV are taking place in the camps. | Apply this code to texts describing that awareness-creation activities on GBV are taking place in the camps. |  | *"They provide education on GBV. They have gathered women together and taught them about GBV. Honestly, they offer advice. They encouraged us to come to them for guidance, and they also provide advice for adolescents."* |
| Existence of referral linkages | Texts mention that referral networks exist for GBV survivors, enabling them to access care and support on time. | Apply this code to texts mentioning that referral networks exist for GBV survivors, enabling them to access care and support promptly. |  | *"Health workers brought me together to the MPHSS clinic, where I received psychological counselling."* |
| Availability of one-stop centre | Texts describing that one-stop centres are available for sexual violence survivors to receive integrated services. | Apply this code to texts describing that one-stop centres are available for sexual violence survivors to receive integrated services. |  | *"We offer healthcare, legal services, and counseling here at the center. All the services are free."* |
| Availability of safe houses and spaces | Texts describing that safe houses/spaces are available for sexual violence survivors to be protected from reattack by the perpetrator. | Apply this code to texts describing that safe houses/spaces are available for sexual violence survivors to be protected from reattack by the perpetrator. |  | *“We have safe zones for girls and women in the camp. When a woman with GBV comes to us, her physical and mental well-being will be protected.”* |
| Delay in receiving care | Texts mentioning that there are delays in receiving care and support for GBV survivors. | Apply this code to texts mentioning that there are delays in receiving care and support for GBV survivors. |  | *“When I asked them to write a referral letter for my friend who was raped, they told me to be patient and wait a bit. They didn’t want her to receive immediate care.”* |
| Lack of confidentiality and privacy | Texts describing that there is a violation of confidentiality, and these issues further hinder survivors' access to care. | Apply this code to texts describing that there is a violation of confidentiality and that these issues further hinder survivors' access to care. |  | *"She may think that her privacy is not being kept secret. She doesn’t want others to know about her situation."* |
| Free services for GBV care | Texts mentioning that services for GBV survivors are available for free. | Apply this code to texts mentioning that services for GBV survivors are available for free. |  | *“The services be legal or health are free.”* |
| Volunteers covering indirect cost | Texts describing that NGOs/volunteers try to alleviate financial hardships by providing financial assistance. | Apply this code to texts describing that NGOs/volunteers try to alleviate financial hardships by providing financial assistance. |  | *"We support survivors with 3,000 birrs for transportation and counselling"* |
| Budget deficiency | Texts mentioning that budget limits impede GBV prevention and response activities. | Apply this code to texts mentioning that budget limits impede GBV prevention and response activities. |  | *“It is challenging to establish awareness for everyone. Educators are sometimes hindered by insufficient funds.”* |
| Normalization of GBV | Texts mentioning that any kind of GBV is perceived as an accepted norm. | Apply this code to texts mentioning that any kind of GBV is perceived as an accepted norm. |  | *"If her husband is always beating her, she may believe he is expressing his love for her."* |
| Blaming the victim | Texts describing that the community blames the GBV survivors. | Apply this code to texts mentioning that the community blames the GBV survivors. |  | *"They say it is your obligation...even though I was the victim, they blamed me."* |
| Preference for mediation | Texts/transcripts mention that the community prefers mediation rather than arresting the perpetrator through legal means. | Apply this code to texts/transcripts mentioning that the community prefers mediation rather than arresting the perpetrator through legal means. |  | *"If both parties agree, mediators can help resolve the situation. Otherwise, she may seek legal assistance."* |
| Community gossip | Texts describing how community gossip about victims prevents them from accessing care and support. | Apply this code to texts describing how community gossip about victims prevents them from accessing care and support. |  | *"If you expose yourself, people will say, 'This is the one who was attacked or raped.'"* |
| Community stigma | Texts describing how the community stigmatizes GBV victims. | Apply codes to texts describing how the community stigmatizes GBV victims. |  | *"Our traditions and norms, the labels given to survivors, discrimination, and stigma—all of these prevent them from receiving care."* |
| Lack of social support | Texts describing the availability of social and community support for survivors. | Apply this code to texts describing the availability of social and community support for survivors. |  | *"I share my condition with the community, but all they do is talk about me. They don’t offer help. I’ve learned from this, so I prefer to go to the health facility on my own."* |
| Availability of community GBV workers | Texts describing that community-based GBV workers are available and have enhanced access to support and care. | Apply this code to texts describing the availability of community-based GBV workers and their enhanced access to support and care. |  | *"Currently, there are women from the IDP community who speak Amharic and are working to address GBV issues."* |
| Overcrowding conditions in the camp | Texts mentioning that overcrowding in the camp significantly impacts access to GBV services. | Apply this code to texts that describe how the overcrowding of the camp drastically affects access to GBV services. |  | *"People in the camp live close together, so if there is any violence, everyone is sure to hear about it. Residents often communicate and share information about what's happening around them, which may lead the survivor to conceal herself."* |
| Security concerns | Texts describing that GBV survivors fear for their safety while going to healthcare or raise security concerns when accessing care and support | Apply this code to texts describing that GBV survivors express fears for their safety when seeking healthcare or raise security concerns while accessing care and support |  | *They avoid going to health facilities due to various challenges they may encounter along the way.* |
| Sloppy legal process | Texts describing that delays in the legal response discourage survivors from accessing care. | Apply this code to texts mentioning that delays in the legal response discourage survivors from accessing care. |  | *"Survivors seeking legal services often face slow and unresponsive procedures."* |
| Lack of trust in the legal system | Texts mentioning that reporting an event will have no serious consequences, or participants mentioning that survivors lack trust in the justice system. | Apply this code to texts mentioning that reporting an event will have no serious consequences, or participants mentioning that survivors lack trust in the justice system. |  | *"It is grab and release; there is no solution."* |
| Weak legal enforcement | Transcripts/texts mentioning that there is insufficient legal enforcement to penalize the perpetrator. | Apply this code to texts mentioning that there is insufficient legal enforcement to penalize the perpetrator. |  | *"The legal system faces challenges in holding perpetrators accountable, and the process is still evolving."* |
