# Supplementary material for: Investigating Gender-based violence against internally displaced women in Debre Berhan, Central Ethiopia: A mixed-methods study using the socio-ecological framework
Source: PLoS One. 2025 Aug 13;20(8):e0329840. doi: 10.1371/journal.pone.0329840 (PMC12349714; doi:10.1371/journal.pone.0329840)
Supplement: Attachment — Submitted filename: Response to Reviewers and editor.docx [file pone.0329840.s008.docx]

I. **Response to editors' comments**

Dear Editor,

We thank you for your comments and the opportunity to revise our manuscript. We have endeavoured to deal with all of the editorial comments, including the addition of 95% confidence intervals, enhanced background context around the study location, and methodological clarification, most particularly around ethical issues and funding statements. All of the revisions have been highlighted in the revised manuscript and discussed in the response table.

We have also answered each reviewer's complaints one by one. We submitted a separate answer for each of the reviews in the tables below. All the modifications for reviewers' comments are likewise highlighted in the updated article and addressed in the response table below.

We appreciate your guidance during the revision process and trust that the manuscript adheres to the publication requirements.

Sincerely,
Kindie Mitiku

On behalf of all authors

| No. | Comment | Response | Where the response |
| --- | --- | --- | --- |
| 1 | Please include 95% confidence intervals (CIs) for your prevalence estimates, as this will significantly improve the reliability of your findings. A summary table of these estimates, rather than relying solely on visual representations, would also be beneficial | Thank you for providing this important remark. We incorporated 95% confidence intervals for prevalence estimates, as well as tables to display prevalence values with CIs rather than pie charts and graphs. | Result section, line 724-813 |
| 2 | The background section would benefit from a clearer explanation of why **Debre Birhan has been a significant location for internally displaced persons** (IDPs). Expanding on the socio-cultural, economic, and political factors that contribute to GBV risks will strengthen your justification for focusing on this area. | Thank you for your critical remark in this area. In the updated text, we explain why our research region has been an important destination for internally displaced people (see paragraph 5). We've also added paragraphs on the socio-cultural, economic, and political aspects that contribute to GBV hazards in these camps (please see paragraph 6 of the amended text). | Background section, line 248-257 |
| 3 | It is crucial to detail the ASIST-GBV questionnaire, including its structure, item types, and psychometric validation within the Ethiopian context. Specifying how qualitative participants were identified and the number of survivors versus key informants will add depth to your methodology. | We appreciate for expressing this important comment. We appreciated this thoughtful input. We have given a detailed explanation of the ASIST-GBV questionnaire's structure, contents/domains, and validation in Ethiopia among refugees. Based on the reviewers' comments, we have also included a table displaying the questionnaire. Furthermore, we have included information on how qualitative participants were identified. The number of survivors vs key informants is also included in the revised manuscript. | Method section, line 520-240 |
| 4 | Addressing Selection Bias: While your study provides valuable insights through its mixed-methods design, the qualitative component may be subject to selection bias due to the purposive sampling approach for recruiting GBV survivors and NGO staff. We suggest that you discuss this potential bias in more detail, outlining its impact on your findings and considering strategies for mitigating such bias in future research. | Thank you for making this important remark. We have added a note detailing selection biases, their implications, the tactics we used, and future advice for researchers. | In the limitation section, line 1342-1348 |
| 5 | Expanding the Discussion: It would strengthen your paper to address how the findings may or may not extend to other internally displaced populations in Ethiopia and in similar settings elsewhere. Discussing factors such as sociocultural, political, and environmental differences will help the reader better understand the broader applicability and any limitations to generalizability. | Thank you for your helpful feedback. We expanded the Discussion section to show how findings may differ across diverse displacement circumstances, highlighting contextual concerns such as social, political, and environmental changes that may limit generalisability. | Discussion section, line 1317-1324 |
| 6 | Verifying Specific Phrases: We noted certain statements in the discussion and conclusions that warrant more cautious interpretation or further evidence. For example: The phrase “Survivors confront several interlinked constraints at all levels of the socio-ecological framework” suggests quantifiable interconnections between barriers, although your data primarily offer qualitative insights without statistical quantification. Please either support this statement with additional evidence or rephrase it more cautiously | Thank you again. We rephrased this comment in the Discussion to better represent the qualitative nature of our findings while avoiding indicating quantitative relationships that were not investigated. | Conclusion of the abstract section, line 226-228  Discussion section  Conclusion section, line 1351 and1352 |
| 7 | Strengthening the connection between all such claims and the data presented or adjusting the language to reflect the exploratory nature of these findings will improve the credibility and precision of your conclusions. | Thank you for pointing this out. We've changed the language in both the abstract and the conclusion to reflect the exploratory nature of our qualitative data while avoiding overstating the interconnectedness of findings | Abstract and conclusion section |
| 8 | Stakeholder-Specific Recommendations: Your conclusion would be strengthened by making recommendations more specific and actionable for different stakeholders. Rather than general suggestions, please provide distinct recommendations for policymakers, NGOs, and camp administrators. | Thank you for your valuable feedback; we have included stakeholder-specific recommendations into the modified draft. | Conclusion and recommendation section, line 1358-1391 |
| 9 | Use precise language throughout, especially when discussing the experiences of GBV survivors. Avoid terms that might imply expertise in trauma, and ensure all statements made in the discussion are supported by your data or cautionary language when required. | We have avoided language that would indicate expertise in trauma. We tried to ensure all comments made in the debate are supported by our data. | All results and discussion section. |
| 10 | You indicated that you had ethical approval for your study. In your Methods section, please ensure you have also stated whether you obtained consent from parents or guardians of the minors included in the study or whether the research ethics committee or IRB specifically waived the need for their consent. | We have added information related to consent from parents or guardians of the children. For study participants under the age of 18 years, the research ethics committee waived parental or guardian permission. This is because GBV is a delicate matter, and engaging parents or guardians may result in unintentional injuries or hazards to GBV survivors. | Method section, line 516-519 |
| 11 | We note that the grant information you provided in the ‘Funding Information’ and ‘Financial Disclosure’ sections does not match. | Fixed as per your remark. |  |
| 12 | Thank you for stating the following in the Acknowledgments Section of your manuscript: “We express our heartfelt appreciation to the consortium of the Guttmacher Institute, Addis Ababa University's School of Public Health, and St. Paul IRHR for their significant support  and funding of this project. We are grateful to the research participants, data collectors, and supervisors for their valuable efforts. Our heartfelt gratitude also goes to the coordinators of internally displaced people for supplying vital background information that aided this project.” We note that you have provided funding information that is not currently declared in your Funding Statement. However, funding information should not appear in the Acknowledgments section or other areas of your manuscript. We will only publish funding information present in the Funding Statement section of the online submission form. Please remove any funding-related text from the manuscript and let us know how you would like to update your Funding Statement. Currently, your Funding Statement reads as follows: “Authors who received each award: KMK  Grant numbers awarded the author:09/2024  The full name of each funder's. ST.PAUL INSTITUTE FOR REPRODUCTIVE HEALTH AND RIGHTS  URL of each funder website:[https://www.spirhr.org/](https://www.spirhr.org/)  Funders role: The funder played a role in the study design, data collection, and analysis but did not provide funding for manuscript preparation and publication”  Please include your amended statements within your cover letter; we will change the online submission form on your behalf. | As directed, we have removed the funding information from the Acknowledgments section of our manuscript. Revise our Funding Statement as below: Authors who won each award: KMK Grant numbers received by the author: 09/2024 Complete names of all funders: St. Paul Institute for Reproductive Health and Rights (SPIRHR), in collaboration with the Guttmacher Institute and School of Public Health at Addis Ababa University Funder's role: The funder supported study design, data collection, and analysis. They did not support manuscript preparation or publication. |  |
| 13 | Please include a copy of Table 3 which you refer to in your text on page 13. | Thank you for your critical insight. We rectified it as per your remark. |  |
| 14 | We note you have included a table to which you do not refer in the text of your manuscript. Please ensure that you refer to Table 4 in your text; if accepted, production will need this reference to link the reader to the Table. | Corrected as per your remark. |  |

II. **Response to Reviewer One’s (Reviewer-1) comments**

| No. | Comments | Response | Where the response is found |
| --- | --- | --- | --- |
| Background |  |  |  |
| 1 | First line, clarify that GBV does not just affect displaced people, but disproportionately does. | We appreciate this. This is an excellent observation. The criticism has been gratefully acknowledged, and a statement in the second paragraph describes how GBV affects displaced persons differently from non-displaced people. We included this in the second paragraph to preserve the logical flow and consistency of paragraphs, as well as to address other reviewers' concerns. | Background section, line 105 |
| 2 | Second paragraph, spell out what IDP is here, instead of in third paragraph where you do now. I would also have a sentence or two defining it. As it is a key point to your article, you want to make sure everyone is on the same page about what it is. | We assumed that readers of this text understood what IDPs are. However, as you correctly pointed out, understanding what IDPs are is critical to ensuring that all readers are on the same page. Thank you for your critical observation. In response to your feedback, we have included a definition of IDPS in the third paragraph. We included the term in the third paragraph to preserve the logical flow and cohesion of the paragraphs. | Background section, line 112 |
| 3 | Third paragraph, clarify what ‘internal disputes’ are | We have replaced the term “internal disputes” with armed conflicts and inter-ethnic clashes | Background section, line 136 |
| 4 | Paragraph five, put GBV before SRH, as GBV is the focus of the article | Corrected as per your comment. | Background section, line 179 |
| Method |  |  |  |
| 1 | Is this really mixed methods if you don’t integrate the findings somehow? I would think more multiple method | In our study, we used a parallel mixed-methods design with quantitative and qualitative components to address different research objectives: the quantitative component estimated the prevalence of gender-based violence (GBV), while the qualitative component investigated the barriers and facilitators to care access among GBV survivors. Although we presented the data individually based on their respective goals, we integrated the results throughout the interpretation and discussion phase to offer a comprehensive understanding of the scope and context of GBV in our study setting. We have now modified the methodology section to better clarify the justification for using a parallel mixed-methods architecture, as well as how conceptual integration was accomplished in the discussion. | Method section, Line 300-305 |
| 2 | I would put study participants before sample size | As per your feedback, we have positioned the study participants ahead of the sample size. | Method section, line 306-310 |
| 3 | More information about how qualitative participants were identified | Thank you for asking for further information about how qualitative participants were identified. We have provided data regarding the identification of the study participants. | Method section, line 377-387 |
| 4 | Of the 16 participants, how many are survivors and how many are key informants | The research included eleven GBV survivors and five key informants. We have incorporated this information in the amended manuscript's sample size section. | Method section, line 333 |
| 5 | Was the questionnaire changed at all after pretesting? | Thank you for expressing this concern. We made a minor change to the questionnaire following the pretest. We included this information in the data gathering section. | Method section, Line 347 |
| 6 | How long was the questionnaire, how long did it take? | Thank you for reminding us to mention the time required to complete the survey. We have put a notice on this (please see the Data collecting tools and methods page). | Method section, Line 349 and 340 |
| 7 | What else was included in the questionnaire besides measuring GBV? | The questionnaire covered respondents' sociodemographic information, categories of GBV, when they happened, and the perpetrators of the violence(kindly refer to the Data gathering methods and procedures part) | Methods section, line 348-350 |
| 8 | How was the ASIST-GBV questionnaire validated in Ethiopia? Include reference, or if it was your team, include details. | Yes, the instrument was validated in Ethiopia in the refugee situation, and it has been utilised in additional research among internally displaced persons in northern Ethiopia. We have supplied references and a full discussion. | Method section, line 412-450 |
| 9 | I’d include the six ASIST-GBV in your description, or make a table | Thank you for reminding us to provide a description or table regarding six ASIST-GBV. we have provided an explanation as well as a table that includes all six questions in Table 1. | Method section, Lines 431 and 432 |
| 10 | Was the same interview guide used for survivors and key informants? | We employed different interview instructions for key informants and GBV survivors, while the main contents are practically comparable. We have erroneously provided just one of the interview rules, which is solely for GBV survivors. Sorry for this accidental error and the difficulty we have caused. Now we have incorporated two distinct interview instructions for key informants and GBV survivors in the amended manuscript(Interview guides 2 and 3) | Method section, line 353 |
| 11 | Break up qualitative and quantitative methods more clearly | We have divided the qualitative and quantitative techniques for improved clarity (please refer to the analysis section of the modified text). | Method section, line 433-473 |
| 12 | Do you mean ‘to ensure no harm was done to GBV SUVIVORS, not sufferers? | It refers to those who have survived gender-based abuse. I apologise for any misunderstandings. We truly appreciated your comment and corrected the grammatical error. | Method section, line 519 |
| Results |  |  |  |
| 1 | Define LARC | The amended paper now includes a comprehensive description of LARC. | Results section line 533 |
| 2 | What all is in the questionnaire? I feel like there needs to be more description of the questionnaire, but since I don’t know what it included, it’s hard to know (please provide the questionnaire as supplementary material) | Thank you for reminding us to include the questionnaire as supplementary material. We have included in the revised manuscript (see additional file 1) | Method section, Line 349 |
| 3 | Have consistency in how you label the participants after quotes. Sometimes you capitalize each word, other times you don’t | We have tried to maintain consistency in labelling the participants after quotes throughout the revised document. Thank you for this important editorial comment. |  |
| 4 | Shouldn’t the structural level come after the community level in the presentation of results? | A nice observation. Yes, as you said, and based on our framework. The structural level should come after the community and even after the institutional level. We addressed this remark in the amended text. | Result section, line 1066-1097 |
| Discussion |  |  |  |
| 1 | Prevalence – you talk about alcohol drinking among IDPs in northern Ethiopia, is that in general or among individuals with GBV? Do you have information on alcohol in your sample? I want to make sure it doesn’t come off as victim blaming | Previous studies treated alcohol intake as a general contextual component, but this was not seen in our sample. To avoid victim blaming, we removed any inference that alcohol use among IDPs in this study was connected with GBV incidence. | Discussion section, Line 1111 |
| 2 | I’d like you to discuss the complication when the perpetrator is family or a person in authority – that really complicates so many aspects and I believe requires specific interventions. I’d like to see more in recommendations – if so much occurs in camps, what specific strategies may be more effective there? Are there examples of initiatives in other locations? | Really, this was a good observation. We have included a discussion of the complications of GBV in case the perpetrators are family or a person in authority. We have also included tailored intervention for such kinds of GBVs. | Discussion section, line 1158-1183 |
| 3 | Also, majority of violence was threatened – what does that really mean? Does that often lead to actual violence? How does that affect stress and well-being? | We have included a description about whether threatened violence leads to actual violence and the complications of it in mental health in the revised manuscript. | Discussion section, Line 1148-1151 |
| 4 | I’d like to see more in recommendations – if so much occurs in camps, what specific strategies may be more effective there? Are there examples of initiatives in other locations? | We have included specific recommendations for camps in the revised manuscript | Conclusion and recommendation section 1358-1362 |
| 5 | What is FGM? | The term FGM is clarified in the modified manuscript | Limitation section, line 1327 |
| 6 | I wouldn’t say age range was a limitation since that was how you designed your study for reproductive | Thank you for highlighting this concern. We see this as a design element rather than a constraint in itself. As a result, we deleted this from the limitations section. |  |
| 7 | Sociological framework – some of the institutions and health system level indicators  don’t have a – or + sign | corrected as per your comment |  |

III. **Response to Reviewer Two’s (Reviewer-2) comments**

| No. | Comments | Responses | Where is it found |
| --- | --- | --- | --- |
| 1 | It is suggested to verify that the keywords are registered as MeSH | As you correctly pointed out, several of our key words are not mesh terminology. Thank you for your critical insight. We acknowledged your suggestion and substituted non-Mesh terminologies with their closest Mesh-approved equivalents. The new keywords are: gender-based violence, health services accessibility, social stigma, social support, patient acceptance of health care, health promotion, Ethiopia. | Abstract section, lines 67 and 68 |
| 2 | It is necessary to briefly define in the background what is meant by gender-based violence in the context of displaced women. | Thank you for your remark. We defined GBV in the opening paragraph of the paper. | Background section, line 97-102 |
| 3 | In methods, it would be pertinent to add a brief explanation about the socio-ecological framework and how its four levels were interpreted in the study variables. | Thank you for reminding us to include explanations about the socio-ecological framework and how we interpreted the four levels in the study variables. We have addressed this valuable comment in the revised manuscript (kindly refer to the qualitative data analysis subsection) | Method section, line 465-472 |

IV. **Response to Reviewer three's (Reviewer-3) comments**

| No. | Comments | Response | Where is it found |
| --- | --- | --- | --- |
| Abstract and background |  |  |  |
| 1 | Author writes: “...unwillingness to reveal their status" - clarify what "status" refers to in this context. Immigration status? Status as a GBV victim? | Very excellent observation! Dear reviewer, we have updated "unwillingness to reveal their status" with "unwillingness to disclose their status as survivors of GBV". | Abstract section, Line 48 |
| 2 | The background section would benefit from a brief explanation of why Debre Birhan specifically has attracted so many IDPs compared to other locations. | Thank you for the suggestion. In the updated paper, we incorporated a paragraph explaining why Debre Birhan is an important area for IDPs in comparison to other places. Kindly refer to paragraphs 4 and 5. | Background section, line 140-149 |
| 3 | Lines 82-84: The sentence "This rising IDP population in Debre Birhan may exacerbate vulnerabilities among women..." uses causal language ("may exacerbate") without establishing the evidence base for this claim. Consider citing previous research demonstrating this relationship. | Thank you for bringing up this vital comment. We've refined our background in this regard to emphasise the well-documented link between displacement and GBV risks, especially in places like Ethiopia, where economic insecurity, overcrowded shelters, and deep-rooted gender inequalities consistently exacerbate vulnerabilities for displaced women and girls. While we couldn't discover studies specifically about Debre Birhan, evidence from similar IDP communities in the Amhara Region significantly supports our views. Overall, have tried to refine the background section to address this critical comment. | Background section, line 150-156 |
| 4 | I would recommend adding a more compelling case for why Debre Birhan specifically deserves research focus compared to other IDP hubs in Ethiopia and/or the African continent. Extant literature exists on GBV in IDP women on the African continent, so what make the current context of focus unique? Essentially, the background mentions Debre Birhan's location and IDP numbers but doesn't establish what makes this setting particularly significant or unique for studying GBV among displaced populations. | We have provided extra lines outlining why Debre Birhan uniquely needs study priority as opposed to other IDP camps (paragraph 7). | Background sections line 193-254 and 150-156 |
| 5 | Explain any distinctive demographic, cultural, or socioeconomic characteristics of Debre Birhan that might influence GBV patterns compared to other IDP settings. | We have included the contextual (demographic, cultural, or economic features of Debre Berhan) that may enhance susceptibility to GBV. | Background section, lines 150-156 |
| 6 | Highlight whether Debre Birhan faces unique resource constraints that could exacerbate GBV risks. | We sincerely appreciated this remark and attempted to address it in paragraph 7. | Background section, lines 154-156 |
| 7 | Note if there are specific governance structures, security arrangements, or service provision models in the Debre Birhan camps that warrant examination. | We have addressed under paragraph 7 | Background section, lines 154-156 |
| 8 | Mention any preliminary observations or previous studies suggesting that GBV patterns in Debre Birhan might differ from national/continental/global trends | In terms of researcher knowledge and early observations, no study has been conducted on GBV patterns and variables influencing GBV survivors' access to care. We have stated explicitly in paragraph 10. The pattern of GBV may vary from other IDPs due to the contextual considerations discussed in paragraph 7. | Background section, lines 193-254 |
| 9 | Clarify whether Debre Birhan represents an understudied region in comparison to more frequently researched IDP settings in Ethiopia | We have clarified whether Debre Birhan represents an understudied location in relation to other often explored IDP situations in Ethiopia or not (please see paragraph 11). | Background section, lines 193-254 |
| 10 | Line 106-107: The author writes: “but there is a lack of studies applying this approach to GBV services among IDPs in Ethiopia”. Which studies do exist and what are their findings? An overview of your literature review should be included in the background section to help map out the gaps in the extant literature and the significance of your work. | Thank you for your critical remark in this area. We added the available studies and their conclusions in the background section (please refer to paragraph 11 of the updated paper). | Background section, lines 193-254 |
| Method section |  |  |  |
| 1 | Line 137: How was the census conducted? | Thank you for asking for more information on how the census was conducted. We have given a full description of how the census was done in the amended paper. | Method section, lines 341-355 |
| 2 | Line 157: What type of items did the “structured questionnaire” consist of? | The questionnaire included respondents' sociodemographic information, types of GBV, when they occurred, and the perpetrators of the violence. We have included this in the revised manuscript under the subsection ‘Data collection tools and procedures’ | Method section, lines 374-376 |
| 3 | Line 189-191: Elaborate on the processing of validating the questionnaire. How did the author(s) come to the conclusion that the tool had great psychological properties and good reliability in the Ethiopian context? | Great appreciation for raising this important comment. The Other reviewers also raised this concern. We have included a detailed elaboration about the tool, specifically about the validation of the tool in Ethiopia in the humanitarian contexts. Kindly refer ‘Measurement of GBV’ subsection of the revised manuscript. | Method section, lines 443-457 |
| Discussion |  |  |  |
| 1 | Line 425: The phrase "…are more experienced in GBV than IDPs" is problematic when discussing trauma and violence - it suggests expertise rather than victimization. Instead, use precise language such as "higher prevalence rates of GBV" or "greater exposure to GBV." | The phrase is rephrased in the modified manuscript | Discussion section, line 1149-1168 |
| 2 | Lines 524-25: The potential of the ASIST-GBV tool resulting in underestimation of GBV should be included in the discussion of plausible reasons for why the prevalence of GBV was lower in the sample compared to previous similar studies (line 420-29). | We have mentioned in both the discussion and limitations (kindly refer to the refined manuscript) | Method section, lines 1167 and 1168 and limitation section, lines 1353-1356 |
| 3 | Several statements make causal assertions without sufficient evidence. For example, lines 427-429 attribute higher GBV prevalence in Northern Ethiopia to "sociocultural norms, a lack of social support, and alcohol drinking" without explaining the evidence base for these causal relationships. | We have revised those statements which have a causal assertion in the discussion, as our study is exploratory. |  |
| 4 | The findings from the current study do not seem particularly novel. The discussion would benefit from a stronger emphasis on the specific, unique contributions of this study to the field. | Thank you for sharing your concern. We believe our findings add to the field in novel ways, particularly by proving camp-based vulnerability, using a socio-ecological framework, and including GBV survivors' narratives. These characteristics give critical new insights that enhance our current understanding. We have also attempted to refine the manuscript to concentrate on the distinctive, unique contributions of this research to the field. |  |
