# Supplementary material for: Investigating Gender-based violence against internally displaced women in Debre Berhan, Central Ethiopia: A mixed-methods study using the socio-ecological framework
Source: PLoS One. 2025 Aug 13;20(8):e0329840. doi: 10.1371/journal.pone.0329840 (PMC12349714; doi:10.1371/journal.pone.0329840)
Supplement: Attachment — Submitted filename: Response to Reviewers.docx [file pone.0329840.s009.docx]

**Response to Reviewers**

Manuscript ID: PONE-D-25-11392R1

We appreciate the academic editor and reviewer 2's complimentary and intelligent remarks and suggestions. Below is a point-by-point discussion on how each issue was handled in the final document.

**Editor/Reviewer Comment 1: Enhanced Definition of GBV in Displacement Setting**

"While your manuscript provides a general definition of GBV, Reviewer 2 correctly identifies a gap in contextualising this definition specifically for displaced women. Please expand your definition in the Background section to explicitly address how GBV manifests in displacement contexts. Consider incorporating guidance from authoritative sources such as the UN High Commissioner for Refugees (UNHCR) to provide a more comprehensive definition.”

Response: We appreciate this important suggestion. We have revised the background section (Paragraph 2, line 77-81) to include a concrete, context-specific definition of GBV among displaced women. The revised paragraph is:

"In the context of displacement, GBV includes any physical, psychological, sexual or economic act that harms women's safety and dignity due to displacement. In displacement situations, GBV can take many forms, such as sexual abuse and exploitation in exchange for basic survival needs, early or forced marriage, denial of access to essential services, violence by intimate partners, state and non-state actors, and workers from humanitarian organisations.”

**Editor/Reviewer Comment 2: Ecological Framework Integration in Conclusions**

"Your study effectively employs the socio-ecological framework throughout the methodology and results. However, please strengthen the conclusions section by explicitly referencing this framework when interpreting your findings. Consider: Organising your concluding remarks around the four levels of the ecological framework (individual, community, institutional, structural)”

Response: We agree with this conclusion and have reworded the conclusions section accordingly. The last paragraph has been extended to discuss findings within the socio-ecological model, emphasising multi-level facilitators and barriers (kindly see the conclusion section, lines 670- 682).
